# Behind the scenes: Centromere-driven genomic innovations in fungal pathogens

**DOI:** 10.1371/journal.ppat.1012080

**Published:** 2024-03-28

**Authors:** Aswathy Narayanan, Md. Hashim Reza, Kaustuv Sanyal

**Affiliations:** Molecular Mycology Laboratory, Molecular Biology and Genetics Unit, Jawaharlal Nehru Centre for Advanced Scientific Research, Bengaluru, India; Vallabhbhai Patel Chest Institute, INDIA

## Introduction

Adverse environmental factors challenge the existence of every living being. In nature, sexual reproduction and meiotic recombination act to generate genetic variations that help organisms thrive in dynamic and unfavorable niches. The fungal kingdom comprises a minimum of 2.5 million eukaryotic species [1]. Fungi possess a remarkable ability to adapt to various selection pressures. The unusual karyotype plasticity exhibited by fungal pathogens plays a significant role as rare stochastic events like chromosomal rearrangements can drive reproductive isolation and increased resistance to antifungal drugs [2,3]. Such events may help predominantly asexual fungal species to remain successful in the evolutionary arms race. Karyotypic rearrangements arising from mitotic recombination events are widely documented in fungi [4]. While we are unaware of the natural frequency of these events, we witness the instances when such a change confers a selective advantage to the organism to adapt to a specific condition.

A growing body of evidence suggests that the centromere locus is one of the significant hubs involved in karyotype diversity, with the potential to stabilize an altered genomic state. Centromere loci, marked by a histone H3 variant, CENP-A^Cse4^, are the primary chromosomal constrictions onto which multiprotein complexes called kinetochores assemble. The centromere-kinetochore nucleoprotein complex provides the platform for spindle microtubules to capture chromosomes and segregate duplicated sister chromatids equally into daughter cells during cell division. In the last few decades, combining genetics, biochemical techniques, next-generation sequencing, and *in silico* approaches, landmark studies helped map centromere DNA loci in more than 60 fungal species [5]. These studies significantly improved our understanding of centromere structure–function relationship from an evolutionary vantage point. While several excellent reviews discuss fungal centromere properties in detail [6–8], here we highlight centromere-associated genomic innovations that often remain unnoticed but facilitate landmark events such as fungal species diversification and the emergence of antifungal drug resistance.

## Centromeres mediate species diversification in the fungal kingdom

Centromere DNA sequences exhibit remarkable structural diversity in the fungal kingdom, differing in size, repeat and retroelement content, presence of heterochromatin, and GC-content [5]. Below, we summarize the various types of genome alterations mediated by centromeres in fungal pathogens.

### Centromere-type transition

**Fig 1:**
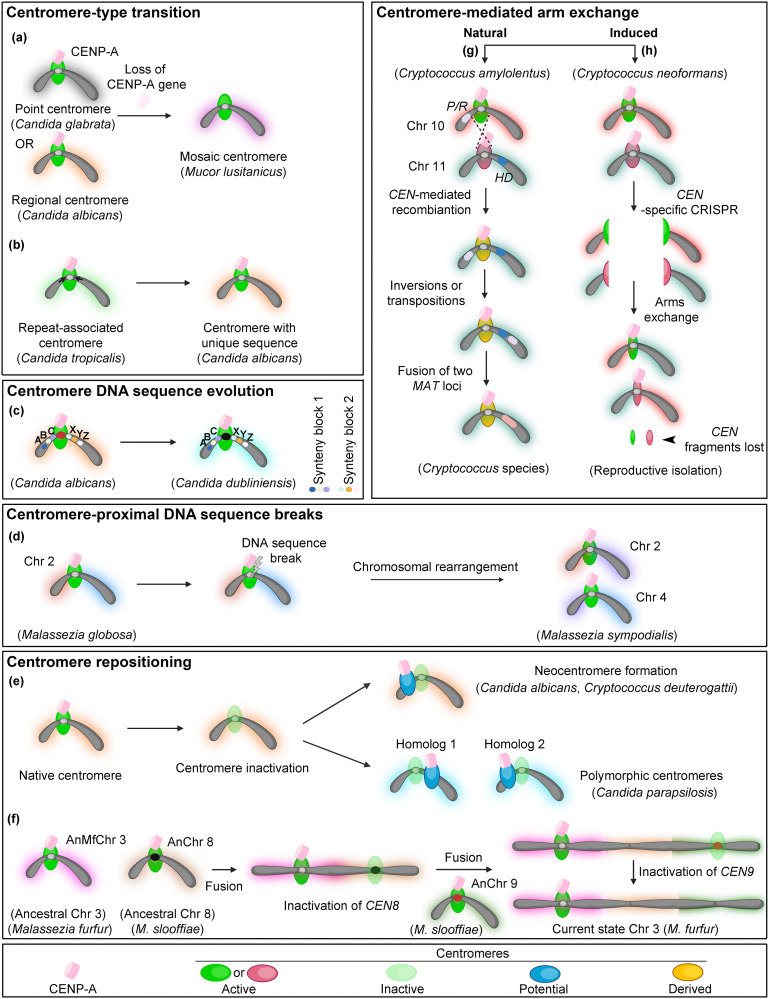
Centromere-associated molecular events contribute to species diversification in fungal pathogens. (a–e) Various molecular events involving centromeres leading to species diversification are depicted, with examples found among the fungal pathogens. (f) An evolutionary model for chromosomal fusion and centromere inactivation in the *Malassezia* species complex is shown. (g) The physiological effects of centromere-mediated mating-type transitions from the tetrapolar system (*HD* and *P/R*) found in environmental species *C*. *amylolentus* to a bipolar system (*MAT*a/*MAT*α) found in pathogenic species such as *C*. *neoformans*. (h) Centromere-mediated chromosome shuffling and recombination lead to reproductive isolation. Created with Biorender.com.

Recent studies have unveiled centromere-type transition events in a few fungal species. Loss of the gene encoding CENP-A^Cse4^ in the genome is associated with the formation of holocentric chromosomes with diffuse kinetochores across the length of the chromosome, in insects like *Bombyx mori* and *Trypanosoma brucei* [9,10]. *Mucor lusitanicus*, belonging to the fungal subphylum of Mucoromycotina, lacking an apparent homolog of CENP-A^Cse4^, possesses monocentric chromosomes with mosaic centromeres combining the properties of point and regional centromeres [11]. Each centromere in *Mucor* has AT-rich, 200-bp core kinetochore-binding regions associated with a centromere-specific sequence motif similar to point centromeres and long, retrotransposon-rich pericentric regions similar to some regional centromeres (Fig 1a).

Another instance of centromere-type transition is observed in the CUG-Ser1 clade of Ascomycota. While *Candida tropicalis* and its related species have regional centromeres associated with homogenized inverted repeats (HIRs), 2 of its close relatives, *Candida albicans* and *Candida dubliniensis*, have centromeres formed on unique, repeat-free DNA sequences [12,13] (Fig 1b). Kinetochore clustering in 3D space in the common ancestor of these species possibly resulted in the proximity of HIRs. Such chromosomal rearrangements were perhaps triggered by occasional chromosome breaks near centromeric regions of homologous DNA sequences leading to centromere-type transition [14].

### Centromere DNA sequence evolution

The centromere paradox states that the centromere DNA sequences evolve rapidly despite their highly conserved function [15]. In *C*. *albicans* and *C*. *dubliniensis*, the gene order or gene synteny around the centromeres remains conserved. However, the centromere DNA sequences of orthologous chromosomes share little or no sequence conservation, suggesting the rapid evolution of the centromere DNA [16] (Fig 1c). Comparing centromere DNA sequences across 4 different geographical clades of *Candida auris* reveals rapid evolution within a species [17]. Similar analyses in closely related species with conserved centromere neighborhoods can further substantiate the rapid evolution of centromere DNA sequences.

### Synteny breaks at centromere proximal sites

Interspecies gene synteny breakpoints map to centromeres in many fungal species complexes and genera like *Malassezia* (Fig 1d), *Verticillium*, and *Candida parapsilosis* [18–20]. Such centromere-proximal chromosomal breakpoints have also been observed in closely related species pairs *C*. *albicans*-*C*. *tropicalis* and *C*. *auris*-*Clavispora lusitaniae* [13,17]. Chromosomal translocations occurring within centromeres, possibly due to the presence of transposable elements, are also known to cause chromosomal variations between *Cryptococcus neoformans* and *Cryptococcus gattii* [21]. In short, DNA sequence features like high AT-content and subsequent secondary structure formation [22], HIRs, retroelements, and spatial proximity resulting from centromere clustering [5,14] are possible underlying factors of such gene synteny breaks and subsequent rearrangements.

### Neocentromere formation upon centromere inactivation

Chromosomal rearrangements like interchromosomal fusions and translocations occasionally bring 2 centromeres on the same chromosome. Dicentric chromosomes, thus formed, are inherently unstable. Different mechanisms of achieving dicentric stability across kingdoms are centromere inactivation, characterized by the selective loss of kinetochore structure, breakage-fusion-bridge (BFB) cycles to obtain monocentric chromosomes, and inactivation of a centromere by transcriptional readthrough or adopting a heterochromatic state [23]. In *Saccharomyces cerevisiae* that possesses sequence-dependent point centromeres, a dicentric chromosome is unstable and undergoes breakage and rearrangements. The chromosome can be stabilized if one of the centromeres can be maintained inactive by transcriptional readthrough or the break–repair cycles result in the physical deletion of a centromere [23]. The mechanisms of inactivation of regional centromeres in fungi remains underexplored.

On the other hand, new centromeres can seed at non-canonical sites once the native centromere function is compromised (Fig 1e). In *C*. *albicans* and *Cryptococcus deuterogattii*, neocentromeres form close to the native centromere locus on its deletion/inactivation and recruit kinetochore proteins [24,25]. Natural neocentromeres identified in *Candida parapsilosis* represent a rare intra-species polymorphism of centromere locations [20] (Fig 1e). In a recent study, 2 loci of the same chromosome have been shown to possess functional centromeres marked by enrichment of CENP-A^Cse4^ in *C*. *parapsilosis* [20]. Another chromosome has a repositioned neocentromere, 30-kb away from the IR-associated centromeric region. In both cases, neocentromeres have no discernible sequence or structural features. The centromere is likely heterozygous, with non-identical loci acting as centromeres on the 2 homologous chromosomes in this organism [20].

### Chromosome number variations due to centromere inactivation

The number of chromosomes (N) usually remain unchanged among closely related species. Occasionally, chromosome number variations are observed within a genus. In the basidiomycetous *Malassezia* species complex, the centromeres are found to be associated with 3-kb long AT-rich sequences. Considering *N* = 9 as the ancestral chromosome number state—(a) breakage of a chromosome at the centromere, followed by the fusion of acentric fragments to other chromosomes; and (b) centromere DNA sequence divergence leading to reduced AT-content resulting in inactivation of a centromere [18]—are likely 2 independent events responsible for the reduction in chromosome number (Fig 1f). Similarly, *Candida lusitaniae* has 8 chromosomes with regional centromeres [26], while the related species *C*. *auris* has 7 chromosomes. A recent study revealed 2 *C*. *lusitaniae* centromere neighborhoods to be present on a single chromosome in *C*. *auris*, out of which 1 centromere was inactivated, possibly due to DNA sequence attrition at this locus [17]. Instances of chromosomal fusions resulting in chromosome number reduction is also reported in the plant pathogen, *Fusarium graminearum* [27], which will serve as a suitable system to study the changes at the centromeres during karyotype evolution.

### Centromere-mediated mating-type transitions

Mating compatibility is regulated by the mating-type loci in fungi. Studies in different *Cryptococcus* species identified mating-type transition through a possible repeat elements-mediated intercentromeric recombination event resulting in a derived state [28]. The recombination event also shifted 2 mating-type loci present on different chromosomes (tetrapolar mating system) onto a single chromosome (bipolar mating system) (Fig 1g), indicating that centromere-associated chromosomal rearrangements can alter the physiological properties in closely related species. A recent comprehensive study on multiple species of the *Malassezia* species complex identified centromere-adjacent breaks and translocations as the driving force of mating-type transitions [29].

### Centromere scission directs reproductive isolation

Centromere-associated molecular events occurring within a species can contribute to reproductive isolation, a prerequisite to speciation. In an artificially induced centromere evolution experimental regime, centromere scission from double-stranded breaks generated using the CRISPR/Cas9 system in *C*. *neoformans* led to complex interchromosomal rearrangements (Fig 1h). Some of the resulting isolates failed to undergo sexual reproduction with the parent strain [30].

## Centromeres and antifungal drug resistance

The treatment of invasive fungal infections is hindered by the limited number of antifungals in clinical use and the emerging resistance to the existing limited antifungal drugs [31]. Ploidy changes are stabilized by the presence of centromeres in the duplicated regions and result in the copy number variations of genes contributing to antifungal resistance.

### Whole chromosome duplication

**Fig 2:**
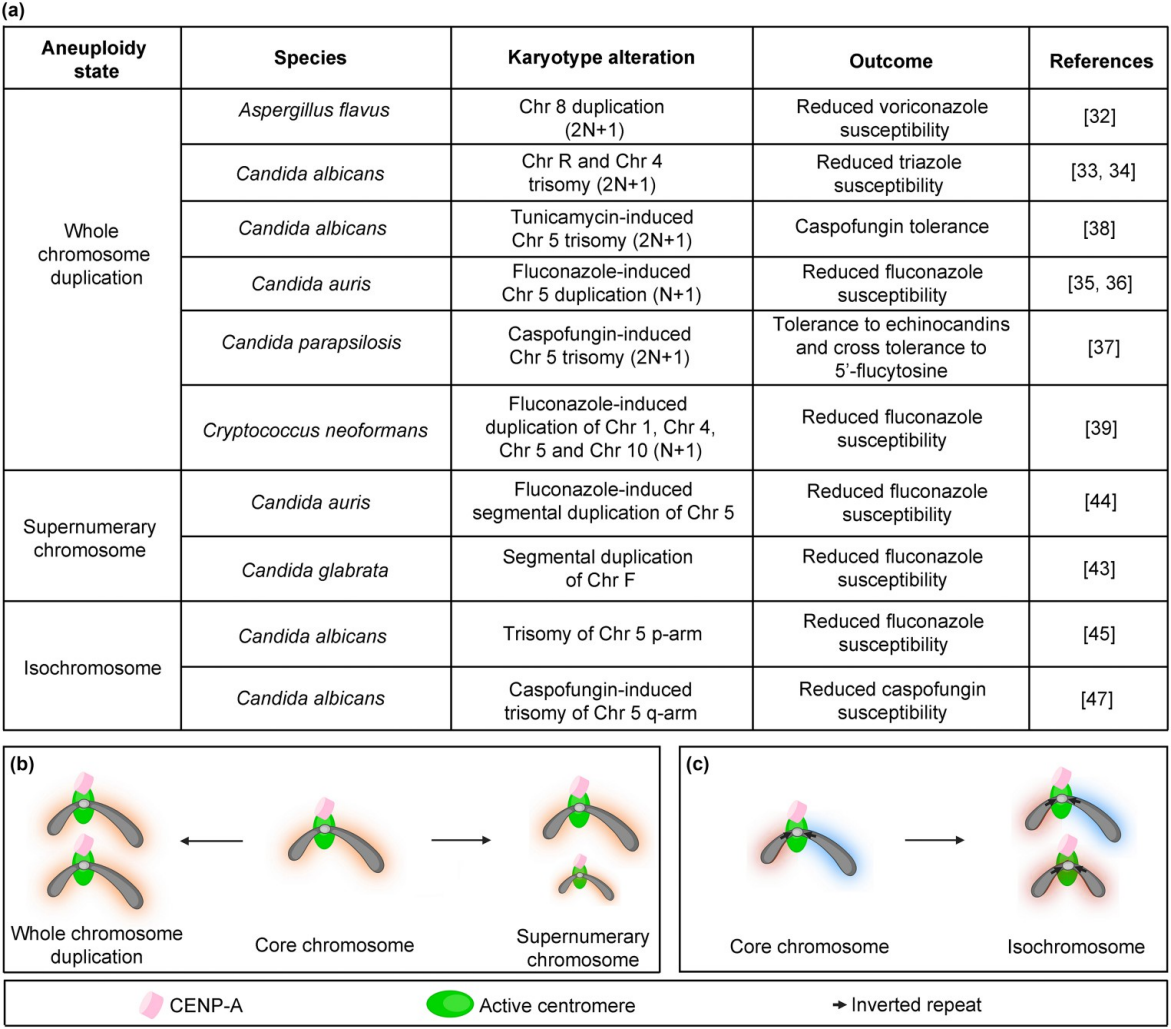
Centromere-stabilized aneuploid states leads to antifungal drug resistance. (a) A table summarizing the outcome of karyotype alterations induced by different antifungal drugs in various pathogenic fungi. (b) Stable aneuploid states existing as whole chromosomes or supernumerary chromosomes arising from the core chromosomes. (c) Isochromosome formation in *C*. *albicans* Chr 5, mediated by centromere-associated inverted repeats. Created with Biorender.com.

Different pathogenic fungal species, under drug pressure, are known to acquire whole chromosome duplications (N+1, 2N+1) that result in reduced drug susceptibility and cross resistance to other drug classes [32–39] (Fig 2a). Molecules involved in general cellular pathways can also influence ploidy—deletion of apoptosis-inducing factor (Aif1) in *C*. *neoformans* causes Chr 1 disomy that underlies improved fluconazole resistance [40]. Aneuploidy (N+1) is also known to be generated for multiple chromosomes during unisexual and heterosexual meiotic reproduction in *C*. *neoformans*, resulting in various phenotypes including fluconazole resistance [41]. In a related species *C*. *deneoformans*, differential expression of some cell-cycle associated proteins were found to orchestrate ploidy changes during unisexual reproduction, with a few segmental aneuploidy conferring azole resistance [42]. Reported aneuploid states associated with anti-fungal drug resistance are discussed below.

### Novel chromosomes

Additional chromosomes arise primarily from 2 major events: segmental duplications and structural rearrangements. Such rearranged chromosomal states are stabilized if they include the centromere DNA sequences and, therefore, may exist as a separate mitotically stable chromosomes [43,44] (Fig 2a and 2b). Centromere-inclusive segmental duplications existing as supernumerary chromosomes are known to confer fluconazole resistance in *C*. *auris* and *C*. *glabrata* [43,44]. An isochromosome formed due to a break at the centromere flanked by inverted repeats followed by a fusion of left arms of the same chromosome was detected in *C*. *albicans* [45] (Fig 2a and 2c). Thus, this isochromosome leads to copy number variations of genes like *ERG11* and *TAC1*, present on the left arm of Chr 5, thereby enhancing azole resistance [46]. Similarly, an isochromosome formed by the right arm of Chr 5 in *C*. *albicans* confers tolerance to caspofungin, belonging to the echinocandin drug class [47]. Conditionally dispensable chromosomes are reported in the plant pathogen, *Zymoseptoria tritici*, wherein centromere properties like the length, lack of repeat elements, and presence of active genes are conserved between core and supernumerary chromosomes. These chromosomes harbor lineage-specific genes that contribute to virulence [48]. Such accessory chromosomes harboring virulence-related genes are also present in the blast fungus, *Magnaporthe oryzae* in which the predicted centromeres are AT-rich, similar to the centromeres of the core chromosomes [49,50].

Remarkably, most of the ploidy changes are induced under antifungal drug stress and are lost during serial passages in drug-free media, resulting in the subsequent loss of acquired drug resistance [43,44], hinting that the strains conditionally maintain the additional chromosomes in such instances.

## Closing comments

The known instances of centromere-mediated karyotype evolution and reproductive isolation do not necessarily reflect the natural frequency of these events. Identifying chromosomal changes in understudied species in different genera, the selective advantages, and their fitness tradeoffs can help us further explore roles of centromeres in evolution.
